# Immediate Effects of Dasatinib on the Migration and Redistribution of Naïve and Memory Lymphocytes Associated With Lymphocytosis in Chronic Myeloid Leukemia Patients

**DOI:** 10.3389/fphar.2019.01340

**Published:** 2019-11-25

**Authors:** Beatriz Colom-Fernández, Anna Kreutzman, Ana Marcos-Jiménez, Valentín García-Gutiérrez, Carlos Cuesta-Mateos, Itxaso Portero-Sainz, Yaiza Pérez-García, Luis Felipe Casado, Fermín Sánchez-Guijo, Joaquín Martínez-López, Rosa M. Ayala, Concha Boqué, Blanca Xicoy, Isabel Montero, César Soto, Raquel Paz, Gabriela Silva, Lorena Vega-Piris, Juan Luis Steegmann, Cecilia Muñoz-Calleja

**Affiliations:** ^1^Servicio de Inmunología, Hospital Universitario de la Princesa, Instituto de Investigación Sanitaria Princesa (IP), Universidad Autónoma de Madrid (UAM), Madrid, Spain; ^2^Servicio Hematología y Hemoterapia, IRYCIS, Hospital Universitario Ramón y Cajal, Madrid, Spain; ^3^Servicio de Hematología y Hemoterapia, Hospital Virgen de la Salud, Toledo, Spain; ^4^Servicio de Hematología y Hemoterapia, IBSAL-Hospital Universitario de Salamanca, Salamanca, Spain; ^5^Servicio de Hematología y Hemoterapia, Hospital Universitario 12 de Octubre, Universidad Complutense de Madrid (UCM), CIBERONC, Madrid, Spain; ^6^Servicio de Hematología Clínica, Hospital Duran i Reynals, Institut Català d’Oncologia, Barcelona, Spain; ^7^Servicio de Hematología, Servicio de Hematología Clínica, Institut Català d'Oncologia, Hospital Germans Trias i Pujol, Barcelona, José Carreras Leukemia Research Institute, Universitat Autònoma de Barcelona, Barcelona, Spain; ^8^Servicio de Hematología, Hospital Universitario Virgen del Rocío, Sevilla, Spain; ^9^Servicio de Hematología, Hospital Povisa, Vigo, Spain; ^10^Servicio de Hematología, Hospital Universitario de la Paz, Madrid, Spain; ^11^Servicio de Hematología, Hospital Universitario de La Princesa, Instituto de Investigación Sanitaria Princesa (IP), Madrid, Spain; ^12^Unidad de Metodología, Instituto de Investigación Sanitaria Princesa (IP), Madrid, Spain

**Keywords:** Dasatinib, lymphocytosis, CCR7, migration, chronic myeloid leukemia

## Abstract

**Introduction:** Dasatinib is a dual SRC/ABL tyrosine kinase inhibitor used to treat chronic myeloid leukemia (CML) that is known to have unique immunomodulatory effects. In particular, dasatinib intake typically causes lymphocytosis, which has been linked to better clinical response. Since the underlying mechanisms are unknown and SRC family kinases are involved in many cell motility processes, we hypothesized that the movement and migration of lymphocytes is modulated by dasatinib.

**Patients, Materials and Methods:** Peripheral blood samples from CML patients treated with second-line dasatinib were collected before and 2 h after the first dasatinib intake, and follow-up samples from the same patients 3 and 6 months after the start of therapy. The migratory capacity and phenotype of lymphocytes and differential blood counts before and after drug intake were compared for all study time-points.

**Results:** We report here for the first time that dasatinib intake is associated with inhibition of peripheral blood T-cell migration toward the homeostatic chemokines CCL19 and CCL21, which control the trafficking toward secondary lymphoid organs, mainly the lymph nodes. Accordingly, the proportion of lymphocytes in blood expressing CCR7, the chemokine receptor for both CCL19 and CCL21, decreased after the intake including both naïve CD45RA+ and central memory CD45RO+ T-cells. Similarly, naïve B-cells diminished with dasatinib. Finally, such changes in the migratory patterns did not occur in those patients whose lymphocyte counts remained unchanged after taking the drug.

**Discussion:** We, therefore, conclude that lymphocytosis induced by dasatinib reflects a pronounced redistribution of naïve and memory populations of all lymphocyte subsets including CD4+ and CD8+ T-cells and B-cells.

## Introduction

Dasatinib is a second generation multitargeted tyrosine kinase inhibitor (TKI) directed against BCR-ABL, which is approved for the treatment of Philadelphia chromosome positive (Ph+) leukemias, including newly diagnosed chronic myeloid leukemia (CML) in the chronic phase (Talpaz et al., 2006; Jabbour and Lipton, 2013). In addition to the BCR-ABL fusion protein, dasatinib inhibits with high potency several receptor tyrosine kinases (RTK) such as c-KIT or platelet-derived growth factor receptors (PDGFR) as well as a broad range of cytoplasmic kinases including TEC, SYK, and SRC family kinases (SFK) (Bantscheff et al., 2007; Rix et al., 2007; Steegmann et al., 2012). SFK are involved in mediating signal transduction from cell surface receptors and regulate fundamental cellular processes including migration, adhesion, invasion, angiogenesis, proliferation, and differentiation (Parsons and Parsons, 2004). Therefore, SFK play a major role in the development, growth, progression, and metastasis of a wide variety of human cancers, and their inhibition by dasatinib can suppress tumor growth and metastatic dissemination of several human cancer cell lines (Montero et al., 2011). Finally, several kinase targets of dasatinib are known to be important in the immune system function. For example, ZAP-70, LCK, FYN, and ITK are essential in T-cell signaling, while LYN, SYK, and BTK play key roles in B-cell receptor signalosome function (Donato et al., 2003; Pene-Dumitrescu and Smithgall, 2010).

The physiological recirculation of lymphocytes between peripheral blood (PB), bone marrow, secondary lymphoid organs (SLO) and other tissues is regulated by a variety of chemokines and their corresponding receptors, which participate in the rolling and firm adhesion phases of the lymphocyte extravasation cascade through the endothelium (Viola et al., 2008; Alon and Shulman, 2011). One of the most important chemokine/receptor pairs is the CCR7 receptor and its ligands, the homeostatic chemokines CCL19 and CCL21, which mediate the main trafficking pathway of T-cells from blood to SLO (Moschovakis and Förster, 2012). Another important pathway in lymphocyte homing to SLO and bone marrow is the CXCR4/CXCL12 axis (Griffith et al., 2014).

Because of its wide inhibition profile, dasatinib inhibits kinases also in healthy normal cells, such as lymphocytes, a mechanism that probably underlies many of the immunomodulatory effects attributed to this drug (Steegmann et al., 2016). Among them, in 2009, Mustjoki et al. reported persistent clonal expansion of cytotoxic T-cells or NK-cells in a distinct group of Ph+ patients receiving dasatinib therapy (Mustjoki et al., 2009; Kreutzman et al., 2010; Nagata et al., 2010; Kreutzman et al., 2011; Powers et al., 2011). Later, the same group showed that dasatinib intake induces a rapid, dose-dependent, and substantial mobilization of lymphocytes in blood peaking 1–2 h after oral intake (Mustjoki et al., 2013). More recently, Paydas and Schiffer et al. investigated, in a large group of patients, the actual incidence of lymphocytosis (Paydas, 2014; Schiffer et al., 2016). They confirmed that lymphocytosis is not observed with other TKI and verified its association with higher response rates and significantly longer response duration. In particular, patients with imatinib-intolerant or imatinib-resistant chronic phase who developed lymphocytosis after treatment with dasatinib had better progression-free and overall survival, suggesting a quite specific immunomodulatory effect of dasatinib.

However, the reasons for the benefit of dasatinib-induced lymphocytosis and its underlying mechanisms are not clear and deserve further research. In this study, we hypothesized that dasatinib may induce lymphocyte mobilization from lymphoid organs or peripheral tissues, it may inhibit their physiological recirculation from the bloodstream to these organs and tissues, or both mechanisms. To our knowledge, there are no reports studying the effects of dasatinib intake on lymphocyte migration *ex vivo*. Therefore, our aim was to study whether the intake of dasatinib changes the migratory pattern of the lymphocytes of the patients as a possible cause of lymphocytosis, to gain insight into the immunomodulatory effect of this TKI.

## Patients and Methods

### Study Patients and Samples

A total of 17 CML patients treated with dasatinib were included in this study. The patients were enrolled in the DASAPOST phase II study (NCT01802450), designed to evaluate the efficacy and safety of treatment change to dasatinib in patients previously treated with first line imatinib who had shown late suboptimal response (patients with complete cytogenetic response (CCyR) without major molecular response (MMR) after at least 18 months of treatment) according to the ELN 09 recommendations. DASAPOST study was approved by the Clinical Research Ethics Committee of Hospital Universitari Germans Trias i Pujol (Badalona, Barcelona, Spain). Patients were treated with dasatinib (Sprycel) 100 mg QD administered orally as continuous daily dosage. A group of healthy donors and a different cohort of CML patients were also included in some experiments. This last cohort included CML patients treated for more than three months with first line or second line dasatinib (100 mg QD) administered orally as continuous daily dosage. The analysis of this second cohort was approved by the Clinical Research Ethics Committee of Hospital Universitario La Princesa (Madrid, Spain) (Reference number PI-561). The study was conducted in accordance with the Declaration of the Helsinki principles. Written informed consent was obtained from each patient and healthy controls prior to sample collection. All measurements of BCR-ABL/ABL levels (international scale, IS) were centralized in a sole EUTOS (European Treatment and Outcome Study for CML) laboratory.

PB samples were obtained the day the patients switched to dasatinib for DASAPOST patients, and at any time point after three months of dasatinib treatment for patients in the other cohort described above. For each patient, two samples were collected: one before the dasatinib dose (preintake sample) and the other one 2 h after drug intake (postintake sample). Only patients having paired preintake and postintake samples were included in the analysis.

Dasatinib used for *in vitro* assays was purchased from Selleckchem, Houston, TX, USA (HPLC purity 99.8%).

### Immunophenotyping

**Figure 1:**
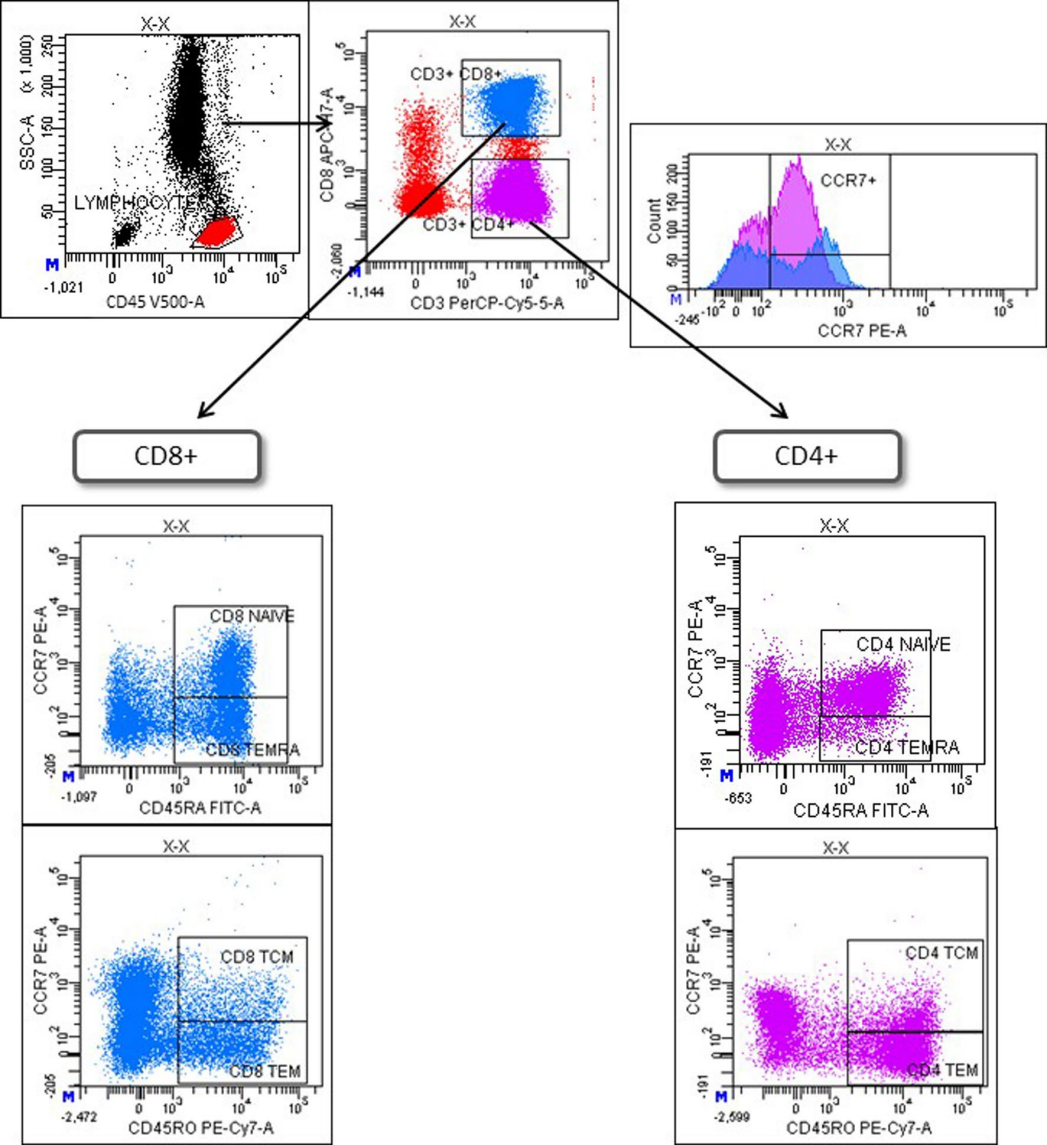
Gating strategy to identify differentiation stages of T-cells. Lymphocytes were selected with a SSC/CD45 gate. Then, CD4+ and CD8+ T-cells were identified as CD3+CD8- or CD3+CD8+ lymphocytes, respectively (upper left). CCR7 expression on CD4+ and CD8+ T-cells is shown in a histogram (upper right). Finally, T-cell maturation stages were defined based on the differential expression of CCR7, CD45RA, and CD45RO on CD8+ (lower left) or CD4+ T-cells (lower right). T_N_ = naïve T-cells (CD45RA+CCR7+); T_CM_ = central memory T-cells (CD45RO+CCR7+); T_EM_ = effector memory T-cells; T_EMRA_ = CD45RA+ effector memory (CD45RA+CCR7-).

Immunophenotyping of preintake and postintake samples was performed on fresh whole blood within 24 h after extraction using the following monoclonal antibodies (mAbs): CD45-V500 (BD; clone HI30), CD3-PerCP (BD; clone SK7), CD8-APC-H7 (BD; clone SK1), CD16-Pacific Blue (BD; clone 3G8), CXCR4 (CD184)-PECy7 (BD; clone 12G5), CD56-Brilliant Violet 421 (Biolegend; clone HCD56), CCR7 (CD197)-PE (RyD; clone 150503), CD27-PE (BD; clone M-T271), CD45RA-FITC (BD; clone HI100), CD45RO-PECy7 (BD; clone UCHL1). Monoclonal isotype controls (IC) were used to define basal levels of immunofluorescence. Whole blood was incubated with the antibodies for 15 min followed by standard lysis and washing steps. A minimum of 100,000 events were acquired with FACSCanto^™^ II flow cytometer and analyzed using FACSDiva software (both from BD Biosciences, USA). First, lymphocytes were identified as CD45+ and then T, B and NK cells were identified as CD3+, CD19+ and CD3-CD16+CD56+ lymphocytes, respectively. CXCR4 and CCR7 expression was evaluated on CD8+ T-cells (CD3+CD8+) and CD4+ T-cells (CD3+CD8-). Finally, naïve (T_N_), central memory (T_CM_), effector memory (T_EM_), and CD45RA+ effector memory (T_EMRA_) T-cells were defined as CCR7+CD45RA+, CCR7+CD45RO+, CCR7-CD45RO+, and CCR7-CD45RA+ T cells, respectively, following the strategy showed in **Figure 1**. CD27 was used to identify naïve CD27- B-cells and memory CD27+ B-cells.

### Migration Assay

PB mononuclear cells (PBMCs) from preintake and postintake samples were separated by Ficoll gradient centrifugation and serum starved in RPMI+0.1% BSA for 30 min. The chemokines CCL19+CCL21 (1 µg/ml) and CXCL12 (0.5 µg/ml) (all PeproTech, Rocky Hills, NJ, USA; SDS-PAGE and HPLC purity ≥ 98%) were added to 24-well plates in a final volume of 600 µl. Polycarbonate filter (5-µm pore size, 6.5-mm membrane, 10-mm thickness, Costar, Cambridge, MA, USA) transwell-inserts were put on top of the wells and starved cells (5x10^5^ cells in 100 µl) were added into the upper chamber of the transwell and were allowed to migrate for 3h at 37°C in 5% CO_2_ atmosphere.

The assay included two control wells: input cells (maximum of cells = 500,000) and basal migration without chemoattractant in the lower chamber. After 3 h, the migrated cells in the lower chamber were collected into tubes and antibodies against CD45, CD3, CD4, CD8, and CD56 were added to each tube. Events were acquired for 210 s by flow cytometry (FACSCanto™ II) and data were analyzed using FACSDiva software. Gates were set as described in the *Immunophenotyping* section.

Two parameters were used to evaluate lymphocyte migratory capacity: the percentage of migrated cell and the migratory index. The percentage of migrated cells was calculated as the ratio between the number of cells migrated in response to CCL19 and CCL21, or CXCL12 and the number of cells present in the input well. The migratory index was defined as the ratio between the number of cells migrated in response to each chemokine and the number of cells migrated into the basal well.

Similarly, *in vitro* effects of dasatinib were analyzed with PBMC from healthy donors, which were pretreated with dasatinib (100 nM) for 1 h and allowed to migrate for 3 h in the presence of the inhibitor.

### Statistical Analyses

Descriptive analysis of the sample: variables were described by their measures of central tendency (mean) and dispersion (standard deviation (SD)) or standard error of the mean (SEM). Normality was tested with Shapiro-Wilk test and homoscedasticity was tested with Levene test. Preintake and postintake samples were compared by using both parametric (paired samples t-test) and nonparametric (Wilcoxon matched-pairs test) methods as appropriate. Adjusted p-value (p-adj) was calculated when an adjustment was necessary due to multiple comparisons (fdr test). P values ≤ 0.05 were considered statistically significant. All analyses were performed using GraphPad Prism version 6 software or R version 3.5.2.

## Results

### Dasatinib Intake Inhibits the Migratory Capacity of T-Lymphocytes

To study the acute effects of dasatinib intake on lymphocyte migration, we obtained samples from 17 CML patients the day they began dasatinib treatment. PB samples were taken before the first drug intake (preintake) and 2 h later (postintake) from all patients. We first studied the acute effects of the first dasatinib intake on PBMC migration by performing a standard transwell assay using CCL19+CCL21 or CXCL12 as chemoattractants. Migrated cells were quantified and phenotyped for the main T lymphocyte subpopulations (CD4+ and CD8+ T-cells) and NK-cells.

**Figure 2:**
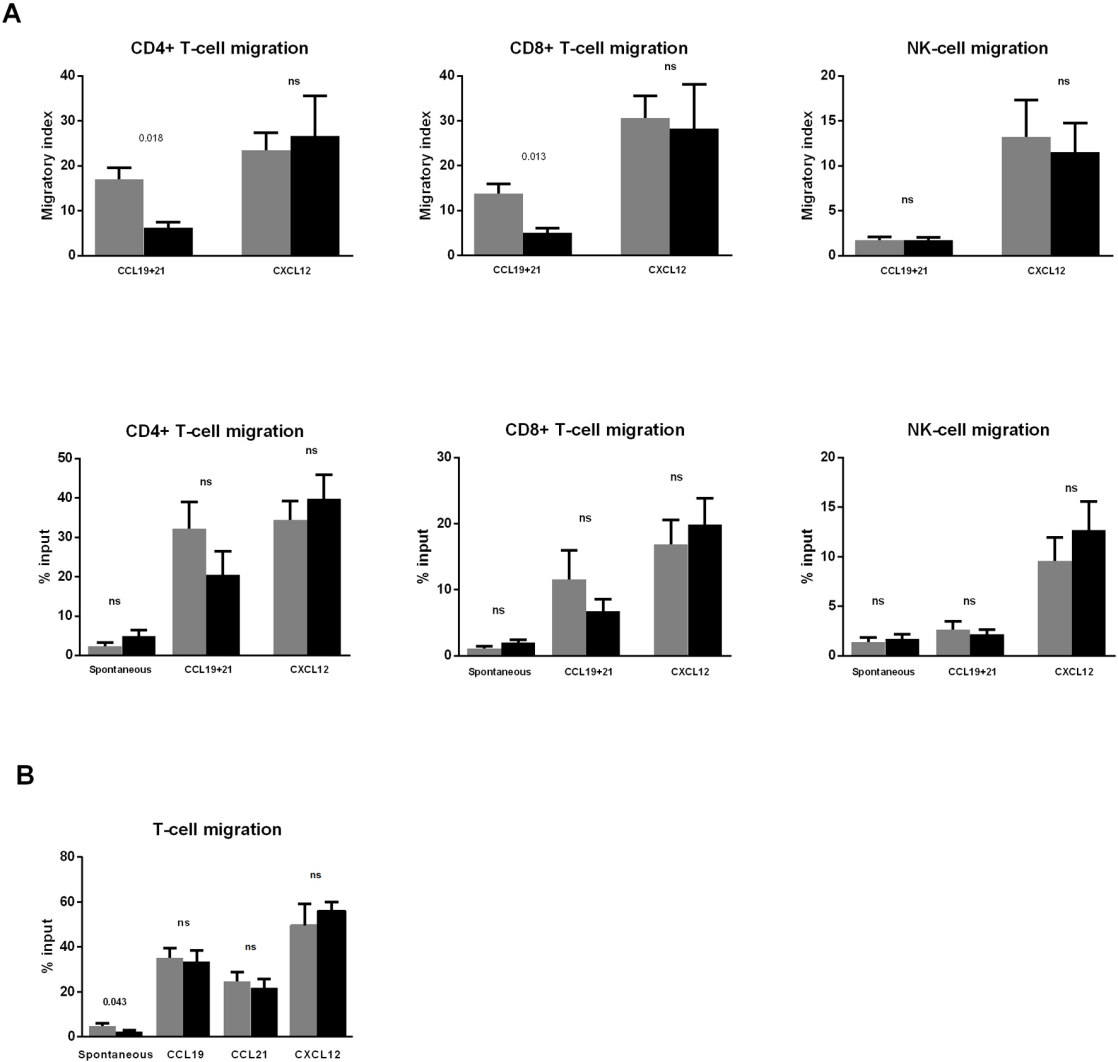
Dasatinib intake reduces CD4+ and CD8+ T-cell migration toward CCL19 and CCL21. **(A)** Blood samples were collected before (pre) and 2 h after (post) the first dasatinib intake in DASAPOST patients. Peripheral blood mononuclear cells (PBMCs) migration was studied by transwell assays, and compared between paired preintake (gray bars) and postintake (black bars) samples from DASAPOST patients (n = 12). The proportion of migrated cells in different cell populations is shown as migratory index (upper panels) and percentage of input (lower panels). **(B)** Migration transwell experiments with healthy donor PBMCs (n = 11) preincubated for 1 h (black bars) or not (gray bars) with dasatinib *in vitro*. Data are represented as means + SEM. Statistically significant p-adj values are shown on the graphs. ns, not significant.

Interestingly, the migratory index toward CCL19+CCL21 was significantly reduced in both CD4+ and CD8+ T-cells (**Figure 2A**, upper panels). A similar tendency was found when the percentage of migration was analyzed, although differences did not reach significance (p = 0.097 for both CD4+ and CD8+ T-cells) (**Figure 2A**, lower panels).

Conversely, the migration of NK-cells toward CCL19+CCL21 was not affected by dasatinib intake. Furthermore, there was no significant effect on the migration of CD4+ T-cells, CD8+ T-cells, and NK-cells toward CXCL12 in postintake samples (**Figure 2A**).

Next, we aimed to analyze whether the inhibition observed in **Figure 2A** was due to a direct effect of dasatinib on the migratory capacity of lymphocytes. To this end, we performed migration experiments with PBMC from healthy donors (n = 11) pretreated *in vitro* with dasatinib for 1 h. This experiment revealed that dasatinib inhibited spontaneous T-cell migration, whereas chemotaxis toward CCL19, CCL21, and CXCL12 was not significantly affected (**Figure 2B**).

### The First Dasatinib Intake Influences CCR7 Expression on T-Lymphocytes

**Figure 3:**
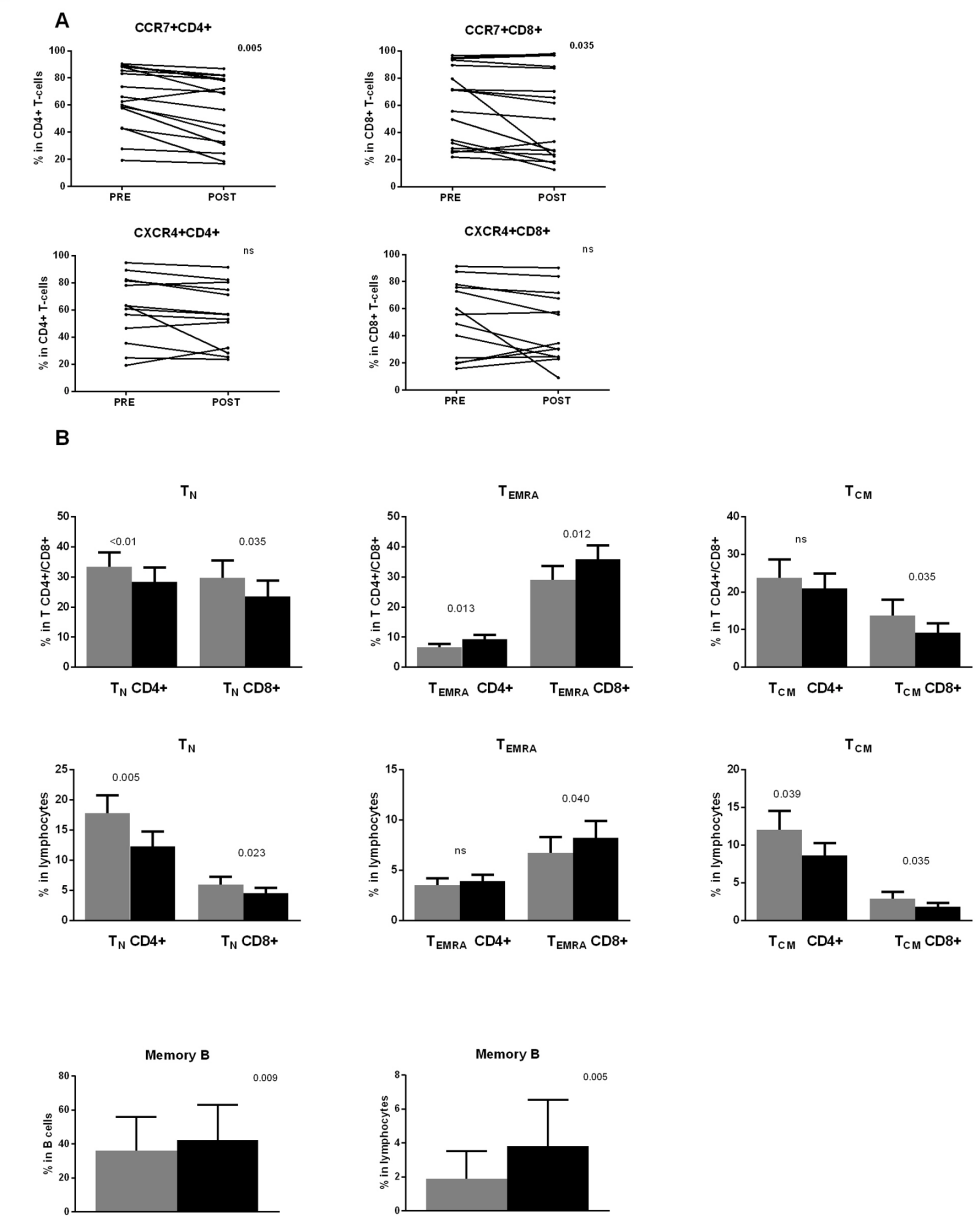
**(A)** The first dasatinib intake changes the proportion of CCR7 positive T-cells. Percentages of CCR7+ and CXCR4+ lymphocytes in CD4+ and CD8+ T lymphocytes are compared between paired samples collected before (PRE) and 2 h after (POST) the first dasatinib intake from DASAPOST patients (CCR7, n = 17;CXCR4, n = 13). Data are represented as dot plots with lines connecting samples corresponding to the same patient. **(B)** T- and B-cell subsets redistribute differently in response to dasatinib therapy. Percentages of naïve (CCR7+CD45RA+)(T_N_), CD45RA+ effector memory (CCR7-CD45RA+)(T_EMRA_) and central memory (CCR7+CD45RO+)(T_CM_) CD4+ and CD8+ T-cells in total CD4+ and CD8+ T-cells (upper panels) and total lymphocytes (middle panels) are compared between paired preintake and postintake samples collected before (gray bars) and 2 h after (black bars) the first dasatinib intake from DASAPOST patients (n = 12). Percentages of memory B-cells in total B-cells and total lymphocytes in the same cohort of patients are shown (lower panels). Data in **(B)** are represented as mean + SEM. Statistically significant p-adj values are shown on the graphs. ns, not significant.

In view of the previous results, an alternative explanation for the findings shown in **Figure 2** could be the presence of a higher proportion of CCR7 negative T-cells in the PB of the patients immediately after dasatinib intake. To confirm this hypothesis, we determined the percentage of CCR7+ and CXCR4+ cells in both CD4+ and CD8+ T-cell subsets and in NK-cells, both in preintake and postintake samples after the first dasatinib dose. This analysis first revealed that the percentage of expression of these chemokine receptors was very heterogeneous among patients before the first dasatinib intake (**Figure 3A**). This variability was already observed in CML patients at diagnosis (data not shown). After dasatinib intake, the percentage of CCR7+ cells in both CD4+ and CD8+ T lymphocytes was significantly lower (**Figure 3A**, upper panels), whereas no significant changes were observed for CXCR4 in both CD4+ and CD8+ T-cells (**Figure 3A**, lower panels). Furthermore, no significant differences were observed in NK-cells (data not shown).

### Dasatinib Favors the Accumulation of Effector T-Cells and Memory B-Cells in Peripheral Blood

CCR7 expression in combination with other molecules defines T-cell maturation stages. After the observation that dasatinib intake significantly decreased the proportion of CCR7+ T-cells, we performed a more detailed phenotypic analysis of lymphocyte subsets according to the expression of CCR7, CD45RA, and CD45RO. We observed an important redistribution of the different subsets within both CD4+ and CD8+ T-cells. In particular, dasatinib clearly increased the proportion of terminal effector CCR7-CD45RA+ T cells (T_EMRA_) in both the CD4+ and CD8+ T-cell subsets whereas in total lymphocytes only CD8+ T_EMRA_ changed (**Figure 3B**, upper and middle panels).

Conversely, the proportion of T-cell subsets expressing CCR7, which include central memory CCR7+CD45RO+ (T_CM_) and naïve CCR7+CD45RA+ (T_N_) T-cells, significantly diminished: CD8+ T_CM_ in both total CD8+ T-cells and total lymphocytes, CD4+ T_CM_ in total lymphocytes, and T_N_ in both CD4+ and CD8+ T-cells and total lymphocytes (**Figure 3B**, upper and middle panels).

Similarly, B-cell differentiation stages were identified by CD27 expression. We observed higher proportions of memory B-cells in total B-cells and total lymphocytes from the postintake samples (**Figure 3B**, lower panels).

Finally, we investigated whether these findings occurred after every drug intake. To this end, preintake and postintake samples from the same patients after 3 and 6 months of treatment and from patients from another cohort treated for more than 3 months with dasatinib, were also analyzed. Similar as to the first drug intake, chemotaxis to CCL19 and CCL21 significantly diminished in the postintake samples of patients previously treated with dasatinib: percentage of input 17% in the preintake sample vs. 10% in the postintake sample (p = 0.0215). Furthermore, we could also confirm a redistribution of maturation stages and an accumulation of CD4+ and CD8+ T_EMRA_ lymphocytes in PB (data not shown).

### A Minority of Patients Do Not Show Increased Absolute Lymphocyte Counts After Dasatinib Intake

**Figure 4:**
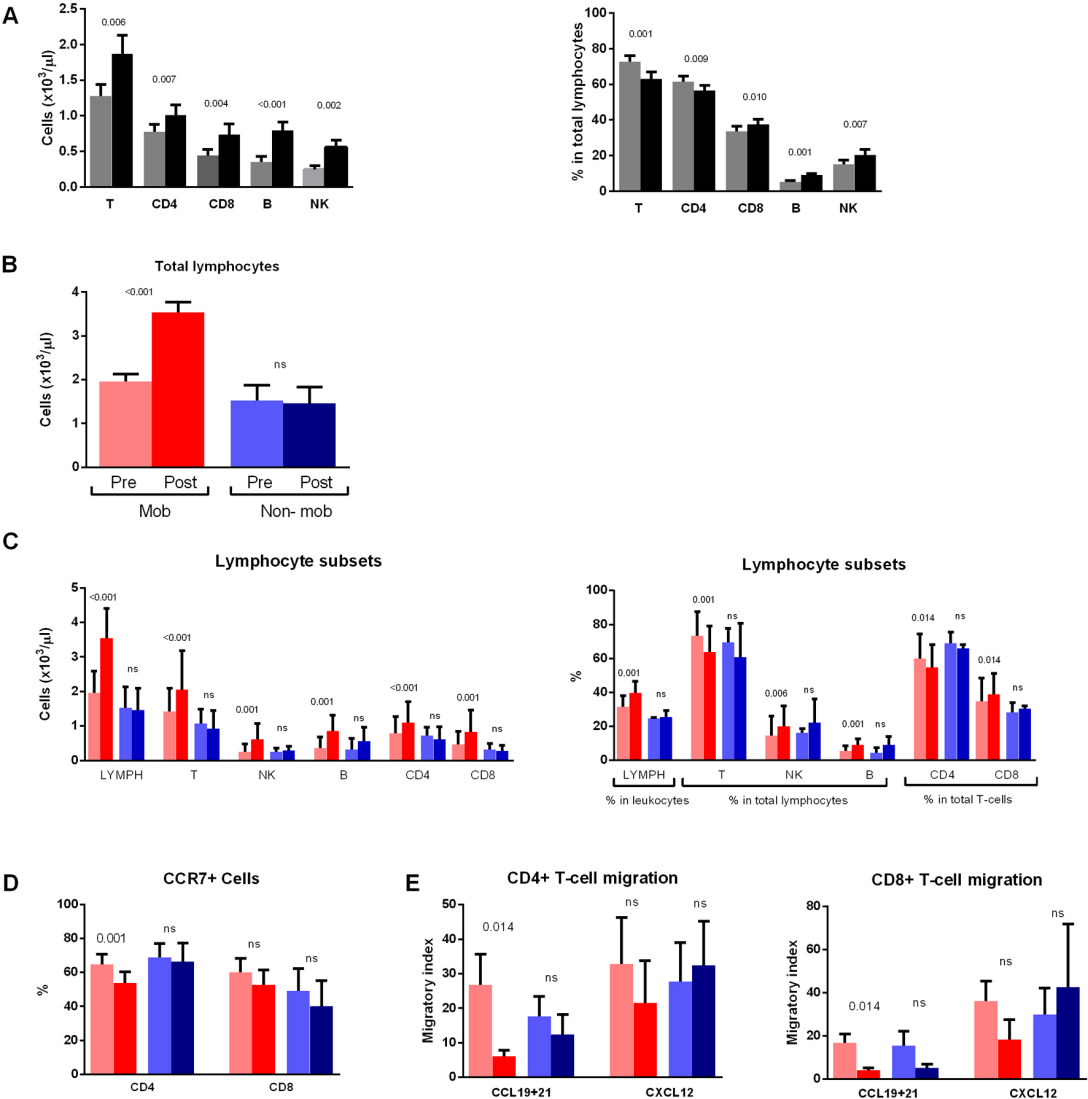
Non mobilizers do not show changes in lymphocyte subsets, chemokine receptor expression and migratory capacity. **(A)** Absolute counts (left panel) and percentages (right panel) of T-, CD4-, CD8-, B- and NK-cells in total lymphocytes are compared between paired preintake and postintake samples collected before (gray bars) and 2 h after (black bars) the first dasatinib intake from DASAPOST patients (n = 17). **(B)** Patients from DASAPOST study were classified as mobilizers (n = 14) or non mobilizers (n = 3) depending on whether their absolute lymphocyte counts increased after the first dasatinib intake or not. **(C)** Absolute counts (left panel) and proportions (right panel) of total lymphocytes, T-cells, NK-cells, B-cells, CD4+ and CD8+ T-cells are compared between paired preintake and postintake samples from mobilizers (n = 14) and non mobilizers (n = 3). **(D)** Expression of CCR7 on CD4+ and CD8+ T-cells is compared between paired preintake and postintake samples from mobilizers (n = 9) and non mobilizers (n = 3). **(E)** Migration of CD4+ and CD8+ T-cells toward CCL19+21 or CXCL12 is compared between paired preintake and postintake samples from mobilizers (n = 9) and nonmobilizers (n = 3). Data are represented as medians+SEM. Statistically significant p-adj values are shown on the graphs. ns: not significant. Red bars, mobilizers; Blue bars: non mobilizers; light colors: preintake samples; dark colors: postintake samples.

The composition of the main lymphoid subsets also changed from the first intake, with a significant increase of absolute counts of B-cells, NK-cells, and T-cells (CD4+ and CD8+) 2 h after the intake (**Figure 4A**, left panel). Furthermore, the proportion of total T-cells and the CD4+ T-cells subset in total lymphocytes decreased in the postintake samples, whereas the proportions of CD8+ T-cells, B-cells, and NK-cells increased (**Figure 4A**, right panel).

Accordingly, absolute lymphocyte counts in blood increased significantly after the first dasatinib intake in most patients. However, we observed that 3 out of 17 CML patients did not have changes in absolute lymphocyte counts in the postintake samples. Therefore, we next divided the patients into "mobilizers" (n = 14) and "non mobilizers" (n = 3) (**Figure 4B**).

The mobilizers had, in addition to increased absolute counts of all lymphocyte subsets in postintake samples (**Figure 4C**, left panel), also increased percentage of total lymphocytes, as well as NK-cells and B-cells (**Figure 4C**, right panel). Conversely, a significant reduction of the proportion of CD3+ T-cells was observed in the postintake samples. Furthermore, the proportion of CD4+ T-cells in total T-cells significantly decreased whereas CD8+ T-cells increased. As expected, no differences in the proportion or absolute numbers of the different populations were observed in those three patients who did not have changes in lymphocyte counts between preintake and postintake samples (**Figure 4C**).

We next analyzed CCR7 expression in these two groups of patients and found that mobilizers had less CCR7+CD4+ T-cells than non mobilizers (**Figure 4D**). A similar tendency was found for CCR7+CD8+ T-cells, although the difference did not reach significance (p = 0.098).

These two groups of patients were compared in terms of the migratory capacity of T lymphocytes, since CCR7 expression on those cells tended to be different between the two groups. Both CD4+ and CD8+ T-cells showed lower migratory index toward CCL19+CCL21 in mobilizers (**Figure 4E**), whereas the migratory index toward CXCL12 was not significantly different between preintake and postintake samples from mobilizers (**Figure 4E**). Interestingly, no differences in CD4+ or CD8+ T-cell migration to any of the chemokines tested were observed in non mobilizers patients after dasatinib intake (**Figure 4E**).

## Discussion and Conclusion

In addition to having potent effects on malignant cells, TKI also target kinases in normal cells, a process that is now known to have consequences in the immune system (Ilander et al., 2014). In this context, dasatinib, a second-generation broad-spectrum TKI, has many unique effects on the immune system such as a well-described rapid, dose-dependent, and substantial increment of lymphocyte numbers in the PB 1–2 h after an oral intake (Mustjoki et al., 2013). Nevertheless, neither the molecular mechanisms underlying this redistribution of lymphoid cells nor its immunological effects have been completely characterized. Other groups have claimed many different reasons to explain the lymphocytosis occurring during dasatinib therapy including viral reactivations and genetic mechanisms (Ilander et al., 2014; Paydas, 2014; Schiffer et al., 2016). However, to our knowledge, this is the first report associating the effects of dasatinib on T-cell migration in CML patients with lymphocytosis. Interestingly, the reduction of the migratory capacity of lymphocytes in response to CCR7 ligands in the post samples of patients is associated with an accumulation of CCR7 negative effector T lymphocytes into PB but not with direct effects of dasatinib on the migration toward CCL19 or CCL21, as we could observe in the *in vitro* experiments. Conversely, we found a significant reduction of lymphocyte spontaneous migration.

In order to confirm whether all these changes underlie the acute lymphocytosis caused by dasatinib, we separately analyzed three non mobilizers patients who did not have changes in the absolute lymphocyte count in the postintake samples and compared them with the mobilizer group of patients, which included most subjects of this study. Interestingly, these non mobilizers did not undergo any of the changes described here, including the redistribution of chemokine receptor expression or the change in the percentage of migration. A limitation of these results is the low number of non mobilizers. The differences are clear and our data are potentially interesting. However, to confirm these results, further studies with a higher number of patients would be required.

Our results therefore suggest that dasatinib-mediated lymphocytosis is partly due to a redistribution and accumulation of terminally differentiated CCR7 negative lymphocytes, both CD4+ and CD8+ T-cells, as well as memory B-cells, in the bloodstream. Moreover, we favor the hypothesis that dasatinib recruits lymphocytes from SLO. Alternatively, this TKI may affect the capacity of terminally differentiated lymphocytes of exiting to the peripheral tissues, causing a lymphocyte blockage on the bloodstream translated into higher absolute lymphocyte counts. The inhibition of spontaneous migration in the *in vitro* experiments supports this last possibility in a scenario where CCR7 positive cells would eventually leave the bloodstream following a chemotactic gradient whereas CCR7 negative lymphocytes would remain temporally "blocked" in PB due to yet undefined effects of dasatinib on motility, adhesion and/or deformability of CCR7 negative lymphocytes.

Still an alternative or additional *in vivo* effect of dasatinib could be the inhibition of chemokine signals required for access of lymphocytes to tissues from the bloodstream, which would lead to a transient accumulation of those lymphocytes and the typical lymphocytosis mediated by this TKI. In addition, dasatinib might block the intimate molecular connection between antigen receptor activation, both in B- and T-cells, and regulation of cell adhesion to other immune cells or to stromal cells through molecules including integrins and chemokine receptors (Carrasco and Batista, 2006; de Gorter et al., 2007; Arana et al., 2008). This is quite intuitive since the immune synapse requires a temporary but stable interaction which is guaranteed by the adhesive connections between the cells involved and the surrounding microenvironment (Martín-Cófreces et al., 2018). In this regard, dasatinib inhibits kinases that are essential in both the B-cell receptor and T-cell receptor signalosomes like LYN, SYK, and BTK, and LCK, ITK, and ZAP-70, respectively (Berg et al., 2005; Chakraborty and Weiss, 2014; Seda and Mraz, 2015). Moreover, it is important to note that some of these signaling modules, including LCK and ZAP-70 are shared by chemokine receptors and the T-cell receptor and that some of the dasatinib targets, e.g., LYN or ITK phosphorylate actin-regulatory proteins like the hematopoietic linage cell-specific protein 1 (HS1). HS1 plays a crucial role as an actin-regulatory protein and, therefore, in lymphocyte cytoarchitecture (Carrizosa et al., 2009). It is likely that the inhibition of all these pathways by dasatinib results in detachment and egress of lymphocytes from the SLO into PB.

This explanation has already been suggested for the mobilization of chronic lymphocytic leukemia or mantle lymphoma cells that occurs with B-cell receptor inhibitors like ibrutinib and fostamatinib (Buchner et al., 2010; de Rooij et al., 2012; McCaig et al., 2012; Chang et al., 2013; Purroy et al., 2017). In addition to these effects, dasatinib also inhibits the metastatic dissemination of solid tumors, an effect which seems to be mediated mainly through the inhibition of SFK (Montero et al., 2011).

Finally, we have recently demonstrated that dasatinib disrupts the homotypic interaction of endothelial cells through phosphorylation of the myosin light chain which, in turn, is probably dependent on the inhibition of SFK (Kreutzman et al., 2017). This effect is reversible but the temporary loss of integrity of the endothelial surface where lymphocytes roll could interfere with their extravasation and explain, at least in part, the temporal accumulation of lymphocytes in the bloodstream.

As we have discussed above, dasatinib could be affecting different signal transduction pathways. At this stage it is unclear which molecular target/s are responsible for the effects of dasatinib described here. However, the identification of these targets is beyond the scope of our study.

Previous reports suggested that dasatinib induces a preferential mobilization of cytotoxic lymphocytes (Mustjoki et al., 2009; Nagata et al., 2010; Kreutzman et al., 2011; Mustjoki et al., 2013). Our results extend those observations and further demonstrate that effector CD4+ T-cells and memory B lymphocytes accumulate after dasatinib intake. These findings suggest that dasatinib is somehow affecting effector/memory lymphocytes independently of their linage. This connects with the interesting field of the different contractile, motile, adhesive, and migratory properties of lymphocytes depending on their maturation stage and/or homing patterns (Jacobelli et al., 2013; Mueller et al., 2013; Martín-Cófreces et al., 2018).

From a clinical perspective, the positive association between lymphocytosis and clinical response (Lee et al., 2011; Paydas, 2014; Schiffer et al., 2016) would be justified not only by the mobilization and accumulation of cytotoxic lymphocytes but also by effector CD4+ T-cells and memory B lymphocytes that we have demonstrated here. It is important to highlight that this rapid mobilization of effector lymphocytes may have prognostic significance and occurs in the majority of patients, whereas a maintained lymphocytosis (defined as 3.6x10^9^/L on at least two consecutive occasions after at least four weeks of treatment) occurs in a moderate percentage of patients treated with dasatinib (Lee et al., 2011; Mustjoki et al., 2013; Schiffer et al., 2016). In addition, we have shown for the first time that this rapid mobilization can be already seen the day the patients begin the treatment and it is likely to occur daily, as indicated by the results obtained in the same patient on the day the treatment begins and several months later. A chronic daily mobilization of effector CD4+ T-helper, cytotoxic CD8+ T-cells, and memory B lymphocytes into tissues infiltrated by the CML, such as PB and bone marrow, may account for the beneficial immunomodulatory effects of dasatinib.

One possible downside of this effect is that lymphocytosis may be associated with both beneficial effects and toxicity of dasatinib. Thus, the incidence of pleural effusion seems to be higher in patients who develop lymphocytosis, although it is only statistically significant in patients with advanced disease (Paydas, 2014; Schiffer et al., 2016; Hughes et al., 2019). A possible explanation to this association is that the extent of disruption of the endothelial cell monolayer (or other stromal cells of the SLO) is related to the magnitude of detachment and egress of effector lymphocytes; either because detachment is a consequence of the disassembly of endothelial cell-cell contacts, or because both effects are caused by the action of the drug on the same target. The increase in permeability would therefore be associated with a greater number of effector lymphocytes in peripheral blood, and both circumstances would favor a pleural effusion with effector lymphocytes, which may explain the exudative nature of the pleural effusion. It should be mentioned that in the largest study of patients receiving dasatinib therapy, advanced age and dose and longevity of treatment were found to be the only risk factors associated to pleural effusion (Hughes et al., 2019). In that sense, the prevalence of effusion with the current 100-mg QD scheme has been considerably reduced with respect to previous schedules, while the mobilization that we have described here seems to occur in the majority of patients with that same scheme. In addition, the lymphocyte mobilization induced by dasatinib seems to be controllable by drug dose (Mustjoki et al., 2013) and recent sub analyses of DASISION trial showed that dose reductions for adverse effects did not affect efficacy. This fact would offer us a therapeutic window to avoid toxic effects on the vascular endothelium but to take clinical advantage of the immediate mobilization of circulating effector and memory cells from all lymphoid subsets.

## Data Availability Statement

All datasets generated for this study are included in the article.

## Ethics Statement

This study was carried out in accordance with the recommendations of Clinical Research Ethics Committee of Hospital Universitari Germans Trias i Pujol (Badalona, Barcelona, Spain) and Clinical Research Ethics Committee of Hospital Universitario La Princesa (Madrid, Spain) (Reference number PI-561). All subjects gave written informed consent in accordance with the Declaration of Helsinki.

## Author Contributions

CM-C, BC-F, AK, VG-G, and JS conceived and designed research. BC-F, AK, AM-J, YP-G, and IP-S performed experiments. BC-F, AK, AM-J and CC-M analyzed data. LV-P performed statistical analysis. BC-F, AK, CC-M, and CM-C interpreted results of experiments. BC-F, AK and AM-J prepared figures. VG-G, LC, FS-G, JM-L, RA, CB, BX, IM, CS, RP, GS, and JS provided study material and patients. CM-C, BC-F, and AK drafted the manuscript. CM-C, BC-F, AK, VG-G, and JS edited and revised the manuscript. All authors approved the final version of manuscript.

## Funding

This work was supported by Bristol Myers Squibb and Grants PI18/01163 from Fondo de Investigaciones Sanitarias to CM-C. CM-C was co-financed by FEDER funds.

## Conflict of Interest

JS and CM-C Have Received Research Funding From Bristol Myers Squibb. The remaining authors declare that the research was conducted in the absence of any commercial or financial relationships that could be construed as a potential conflict of interest.
